# Comparison of accuracy and early outcomes in robotic total knee arthroplasty using NAVIO and ROSA

**DOI:** 10.1038/s41598-024-53789-4

**Published:** 2024-02-08

**Authors:** Masahiro Hasegawa, Shine Tone, Yohei Naito, Akihiro Sudo

**Affiliations:** Department of Orthopaedic Surgery, Mie University Graduate School of Medicine, 2-174 Edobashi, Tsu City, Mie 514-8507 Japan

**Keywords:** Medical research, Rheumatology

## Abstract

This study aimed to compare the cutting and component placement accuracies and early outcomes after total knee arthroplasty (TKA) between an image-free handheld robotic system (NAVIO) and a radiography-based robotic system (ROSA). This retrospective study included 88 patients (88 knees) who underwent TKA using the NAVIO (40 patients) or ROSA (48 patients) robotic systems. The accuracies of the robotic systems were compared. Clinical scores were evaluated using the Knee Society Score 2011 (KSS 2011) and the forgotten joint score (FJS)-12 at 1 year postoperatively. The femoral sagittal cutting error was smaller in the NAVIO group than in the ROSA group. The other cutting errors were not statistically different in both groups. Implantation errors did not differ between the groups. Regarding the clinical outcomes of the KSS 2011 subscales, the symptoms score was higher in knees operated using ROSA than in those using NAVIO. The other KSS 2011 subscales and the FJS-12 showed no differences between the two groups. In conclusion, the femoral sagittal cutting error was smaller in the NAVIO group than in the ROSA group, and the KSS 2011 symptom score subsection at one year was higher in the knees operated using ROSA than in those using NAVIO.

## Introduction

Total knee arthroplasty (TKA) using a robotic system yields excellent knee alignment and reduces radiographic outliers. However, there is a debate surrounding the clinical outcomes after TKA using a robotic system versus the standard TKA^1–4^. Most previous studies have focused on computed tomography (CT)-based robotic systems^1–4^. However, surgeons can perform robot-assisted TKA without preoperative CT imaging^1,5–8^.

**Figure 1:**
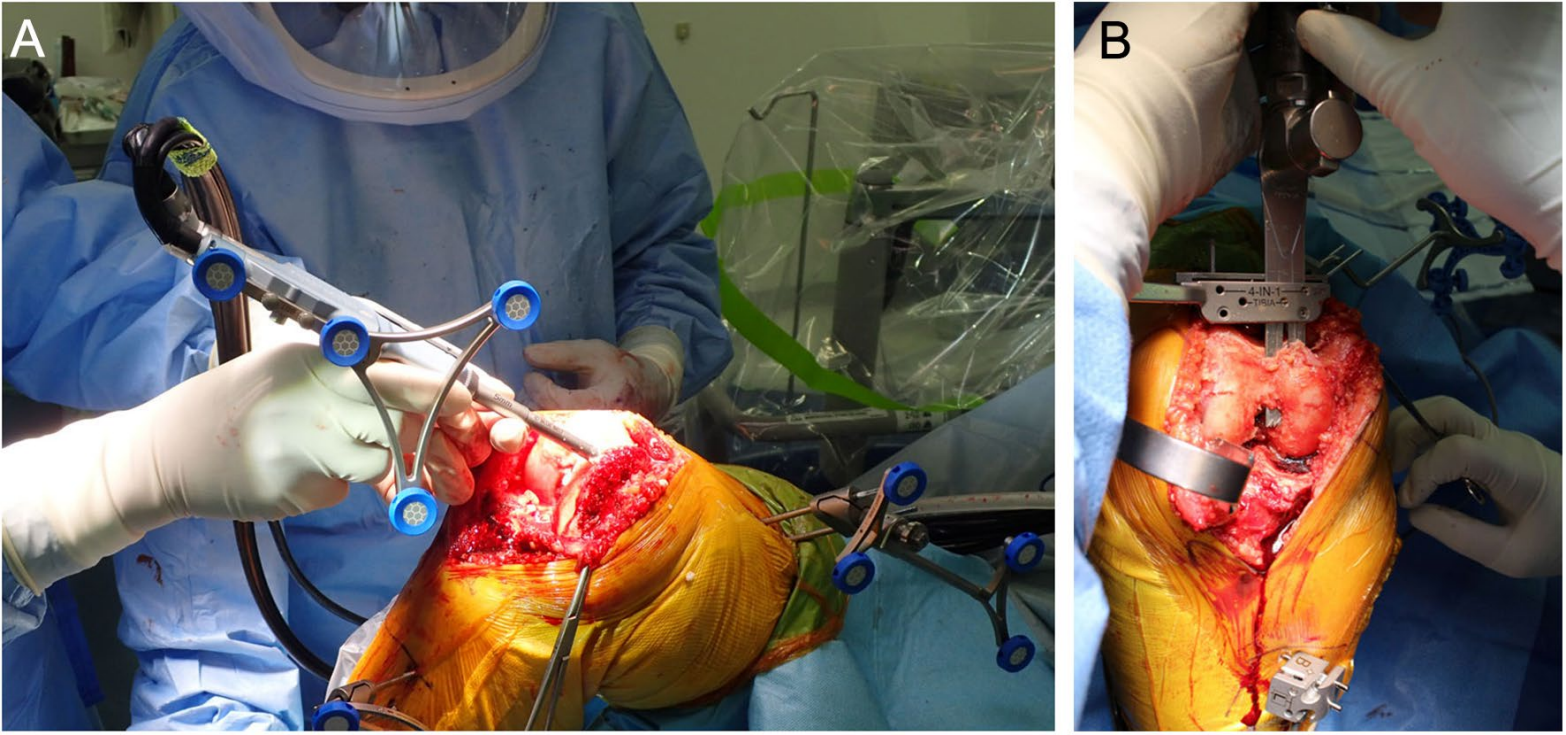
(**A**) NAVIO, cutting the distal femur with a hand-held burring device. (**B**) ROSA, cutting the distal femur using a bone saw.

NAVIO is an image-free handheld robotic system (Smith & Nephew, Memphis, TN, USA). After morphing the femoral and tibial surfaces, surgeons can manually apply varus and valgus stresses during full range of motion (ROM) to apply tension the lateral and medial structures. All cutting levels are determined based on soft tissue laxity. The distal femur and proximal tibia are cut using a handheld burring device with robotic control of the planned resection area and depth (Fig. 1A)^1,5–8^.

Rosa is an image-based robotic system (Zimmer Biomet, Warsaw, IN, USA), where the surgeon can proceed only if the landmark points show a correspondence with the preoperative radiographic planning; it is also available in an imageless option, relying solely on the correct quality of the acquisition for bone morphology and resections^9,10^. As with NAVIO, varus and valgus stresses are applied manually during full ROM, and the cutting levels of the distal femur and proximal tibia are determined based on soft tissue laxity. After intraoperative planning, the ROSA robotic system places and holds the cutting guide at the desired location, and the surgeon cuts the bone through the guide using a bone saw (Fig. 1B)^11,12^.

Both robot-assisted surgical systems enable surgeons to obtain excellent radiographic results and satisfactory early outcomes^7,8,13–15^. Mancino et al.^10^ conducted a comparison between the accuracy of the planned implant positioning of the ROSA robotic system and an accelerometer-based navigation system. The knees treated with ROSA exhibited a significantly reduced error from the planned target angles for both femoral and tibial components. However, no studies have compared the radiographic and clinical outcomes of the NAVIO and ROSA systems.

This study aimed to compare the cutting and component placement accuracies, as well as early outcomes after TKA using NAVIO and ROSA. It was hypothesized that radiographic and early clinical outcomes would be similar when using either NAVIO or ROSA.

## Materials and methods

### Patients

This retrospective study included 88 patients (88 knees) who underwent TKA using the NAVIO or ROSA robotic systems. Forty patients were treated using NAVIO between January 2021 and January 2022, whereas forty-eight patients were treated at another institution using ROSA between February 2021 and December 2021. The exclusion criteria were revision surgeries and severe deformities requiring a constrained implant. The same surgeon (MH) performed all surgeries via the midvastus approach. Table 1 shows the patient demographics including age, sex, diagnosis, and body mass index (BMI).

**Table 1:** Demographic characteristics.

	NAVIO group^7^	ROSA group	p-value
Age (years)	72.3 ± 7.3	73.6 ± 6.4	0.494
Sex			0.131
Male	13	8	
Female	27	40	
Diagnosis			> 0.999
Osteoarthritis	40	47	
Rheumatoid arthritis	0	1	
BMI (kg/m^2^)	27.7 ± 5.1	26.3 ± 5.1	0.081

*BMI* body mass index.

Values of age and BMI are presented as mean ± standard deviation.

### Surgical technique and component design

The anterior and posterior cruciate ligaments were sacrificed in all cases. In the NAVIO group, a second-generation bicruciate-substituting (BCS) prosthesis (Journey II, Smith and Nephew) was used. The surface of the tibial polyethylene insert is concave on the medial side and convex on the lateral side. The coronal joint line was designed with a 3° varus to replicate the normal knee anatomy^16^. In the ROSA group, the Persona Knee (Zimmer Biomet) with a medially congruent (MC) polyethylene insert was used. The MC polyethylene insert has a greater medial contact area to reduce contact stresses and a higher anterior lip on the medial side to increase subluxation resistance^17^. On the lateral side, reduced posterior conformity and an arcuate-bearing path facilitate external rotation. The surface of the insert is concave on both sides.

**Figure 2:**
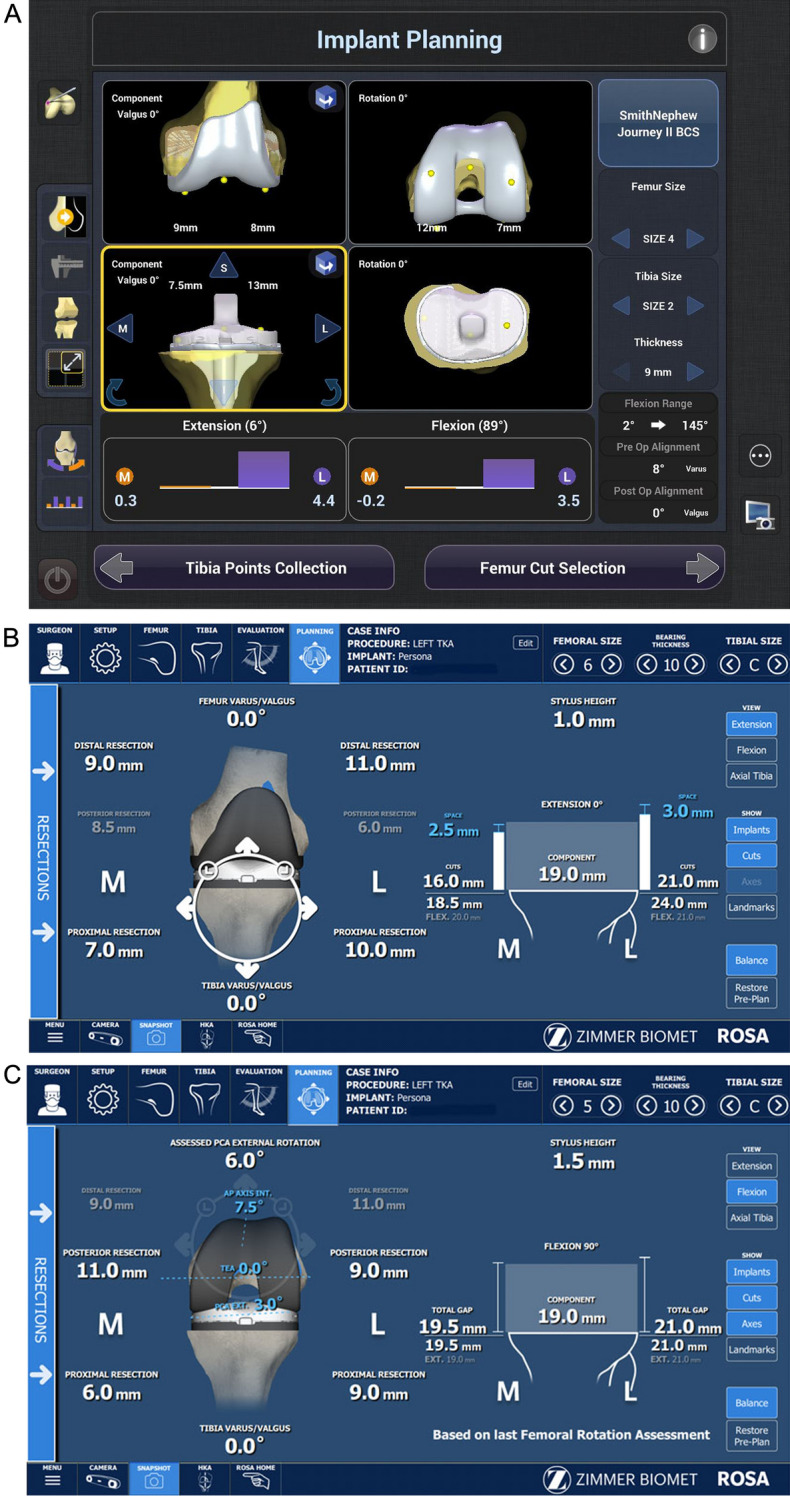
Screenshot of planning for bone cutting. The amount of bone cutting was determined to achieve equal soft tissue balance in extension and 90° of flexion, allowing medial tightness. (**A**) NAVIO, the bone-cutting plan in extension and 90° flexion. (**B**) ROSA, the bone-cutting plan in extension. (**C**) ROSA, the bone-cutting plan in 90° flexion.

In both groups, after applying varus and valgus stresses manually from extension to flexion, the bone cut thicknesses of the distal femur and proximal tibia were planned, allowing for medial tightness (Fig. 2A,B). The distal femoral and proximal tibial osteotomies were planned perpendicular to the mechanical axis in the coronal plane. The flexion angles in the sagittal plane were 3° and 4° in the NAVIO and ROSA groups, respectively. The posterior slopes in the sagittal plane were 3° and 4° in the NAVIO and ROSA groups, respectively. In the ROSA group, the size and external rotation of the femoral component were determined using a tension meter in extension and 90° flexion (Fig. 2C). Femoral rotation was determined to be parallel to the epicondylar axis. In knees with excessive medial tightness, external rotation of the femoral component up to 7° relative to the posterior condylar axis was planned.

### Evaluation

The cutting angles were validated using the robotic system in both groups. After inserting the polyethylene surface, the soft tissue balance was manually evaluated under varus and valgus stresses from extension to flexion. Intraoperative mediolateral laxity was defined as the difference between varus and valgus stresses evaluated at 0°, 30°, 60°, 90°, and 120°.

The cutting errors, intraoperative robotic records, and postoperative measurements obtained using plain radiographs were evaluated. The cutting error was defined as the difference between the planned and validated cutting angles. The implantation error was defined as the difference between the planned and measured radiographic angles. The signed and absolute errors were computed. Positive values in the signed errors indicate valgus in coronal alignment, flexion in femoral sagittal alignment, and posterior tilt in tibial sagittal alignment. Conversely, negative values signify varus in coronal alignment, extension in femoral sagittal alignment, and anterior tilt in tibial sagittal alignment.

After all the components were implanted, the lateral and medial laxities of the knee were manually measured with the knee at 0°, 30°, 60°, 90°, and 120°, guided by the robotic systems. Factors affecting patient satisfaction and expectations, including lateral and medial laxities, were evaluated using the Knee Society 2011 score (KSS 2011)^18^. Clinical scores were evaluated using the KSS 2011 and the forgotten joint score (FJS)-12 at one year postoperatively.

All procedures were performed in accordance with the principles of the Declaration of Helsinki. Our institutional review board approved this study (H2018-083). Prior to enrollment, written informed consent was obtained from each patient.

### Statistical analyses

In previous studies^7,19^, the difference in the femoral sagittal cutting errors between NAVIO and ROSA was 0.4°. Based on this finding, a sample size of 20 knees in each group was required to detect a significant difference between the groups (ɑ = 0.05, power = 0.8).

The age and BMI of the patients, operative time, ROM, KSS 2011, FJS-12, cutting errors, implantation errors, and hip-knee-ankle (HKA) angle were compared between the groups using the Mann–Whitney *U* test. The chi-square test was used to compare the sexes. Preoperative and postoperative comparisons of knee ROM and KSS 2011 scores were performed for each group using the Wilcoxon signed-rank test. Correlation analyses between the laxities (medial, lateral, and mediolateral) and the KSS 2011 categories (symptoms, patient satisfaction, expectations, and functional activities) were performed using Spearman’s rank correlation test in both groups. Statistical significance was set at p < 0.05, using EZR version 1.61^20^.

## Results

The cutting errors are listed in Table 2. The femoral sagittal absolute error was smaller when using NAVIO than when using ROSA. The femoral cutting in the ROSA group exhibited a significantly greater degree of varus angulation and extension. The other cutting errors were not significantly different in both groups.

**Table 2:** Signed and absolute cutting errors.

	Signed error^a^			Absolute error		
	NAVIO group	ROSA group	p-value	NAVIO group^7^	ROSA group	p-value
Femur						
Coronal (°)	0.12 ± 0.44	− 0.07 ± 0.36	0.011	0.36 ± 0.27	0.29 ± 0.22	0.189
Sagittal (°)	0.59 ± 0.45	− 0.87 ± 0.60	< 0.001	0.59 ± 0.45	0.88 ± 0.58	< 0.001
Tibia						
Coronal (°)	0.10 ± 0.44	0.14 ± 0.38	0.718	0.37 ± 0.24	0.33 ± 0.23	0.452
Sagittal (°)	0.07 ± 0.51	− 0.08 ± 0.66	0.453	0.39 ± 0.32	0.55 ± 0.37	0.054

Quantitative variables are presented as mean ± standard deviation.

^a^Positive values indicate valgus in coronal alignment, flexion in femoral sagittal alignment, and posterior tilt in tibial sagittal alignment.

Negative values indicate varus in coronal alignment, extension in femoral sagittal alignment, and anterior tilt in tibial sagittal alignment.

Table 3 displays the implantation errors. No differences were found in the absolute implantation errors between the two groups. However, the femoral component in the ROSA group was implanted significantly more extended in position. HKA angle outliers were not observed between the groups.

**Table 3:** Signed and absolute implantation errors.

	Signed error^a^			Absolute error		
	NAVIO group	ROSA group	p-value	NAVIO group^7^	ROSA group	p-value
Femur						
Coronal (°)	− 0.01 ± 0.64	− 0.14 ± 0.79	0.388	0.41 ± 0.48	0.47 ± 0.65	0.978
Sagittal (°)	− 0.20 ± 1.20	− 1.09 ± 0.89	< 0.001	0.90 ± 0.80	1.11 ± 0.75	0.217
Tibia						
Coronal (°)	− 0.10 ± 0.67	− 0.03 ± 1.48	0.655	0.43 ± 0.53	0.59 ± 1.35	0.996
Sagittal (°)	− 0.51 ± 0.64	− 0.75 ± 0.77	0.117	0.64 ± 0.51	0.90 ± 0.59	0.056

Quantitative variables are presented as mean ± standard deviation.

^a^Negative values indicate varus in coronal alignment, extension in femoral sagittal alignment, and anterior tilt in tibial sagittal alignment.

In the knees operated using NAVIO, the mean ROM significantly improved from 110.5 ± 21.1° to 128.6 ± 13.1° (p < 0.001)^7^. The mean ROM also improved from 114.5 ± 22.6° to 127.3 ± 15.0° in the knees operated using ROSA (p < 0.001). Preoperative and postoperative ROM were not significantly different between the groups (p = 0.331 and p = 0.942, respectively). The preoperative and postoperative KSS 2011 scores are shown in Table 4. Overall scores improved significantly. Category scores for symptoms, satisfaction, and functional activities improved significantly. However, expectation scores worsened (p < 0.001). Regarding the clinical outcomes of the KSS 2011 subscales at one year, the symptoms score subscale was higher in knees operated using ROSA than in those using NAVIO (p = 0.0167), with the other subscales showing no significant differences between the groups. Similarly, postoperative FJS-12 showed no difference between the groups (NAVIO group 66.1 ± 27.4, ROSA group 64.5 ± 22.6, p = 0.566).

**Table 4:** Preoperative and postoperative Knee Society Score 2011.

	Preoperative			Postoperative		
	NAVIO group^7^	ROSA group	p-value	NAVIO group^7^	ROSA group	p-value
Overall	82.2 ± 29.3	85.4 ± 25.8	0.546	115.3 ± 35.0	127.3 ± 27.8	0.075
Symptoms	10.4 ± 6.1	9.1 ± 5.9	0.318	19.9 ± 3.7	21.5 ± 3.8	0.017
Satisfaction	14.5 ± 6.9	14.9 ± 6.1	0.946	24.7 ± 9.5	27.7 ± 8.0	0.166
Expectations	13.7 ± 1.6	13.5 ± 1.7	0.651	8.9 ± 2.8	9.8 ± 2.8	0.164
Functional activities	43.8 ± 21.1	47.9 ± 18.6	0.388	61.8 ± 24.3	69.2 ± 17.8	0.179

Quantitative variables are presented as mean ± standard deviation.

**Figure 3:**
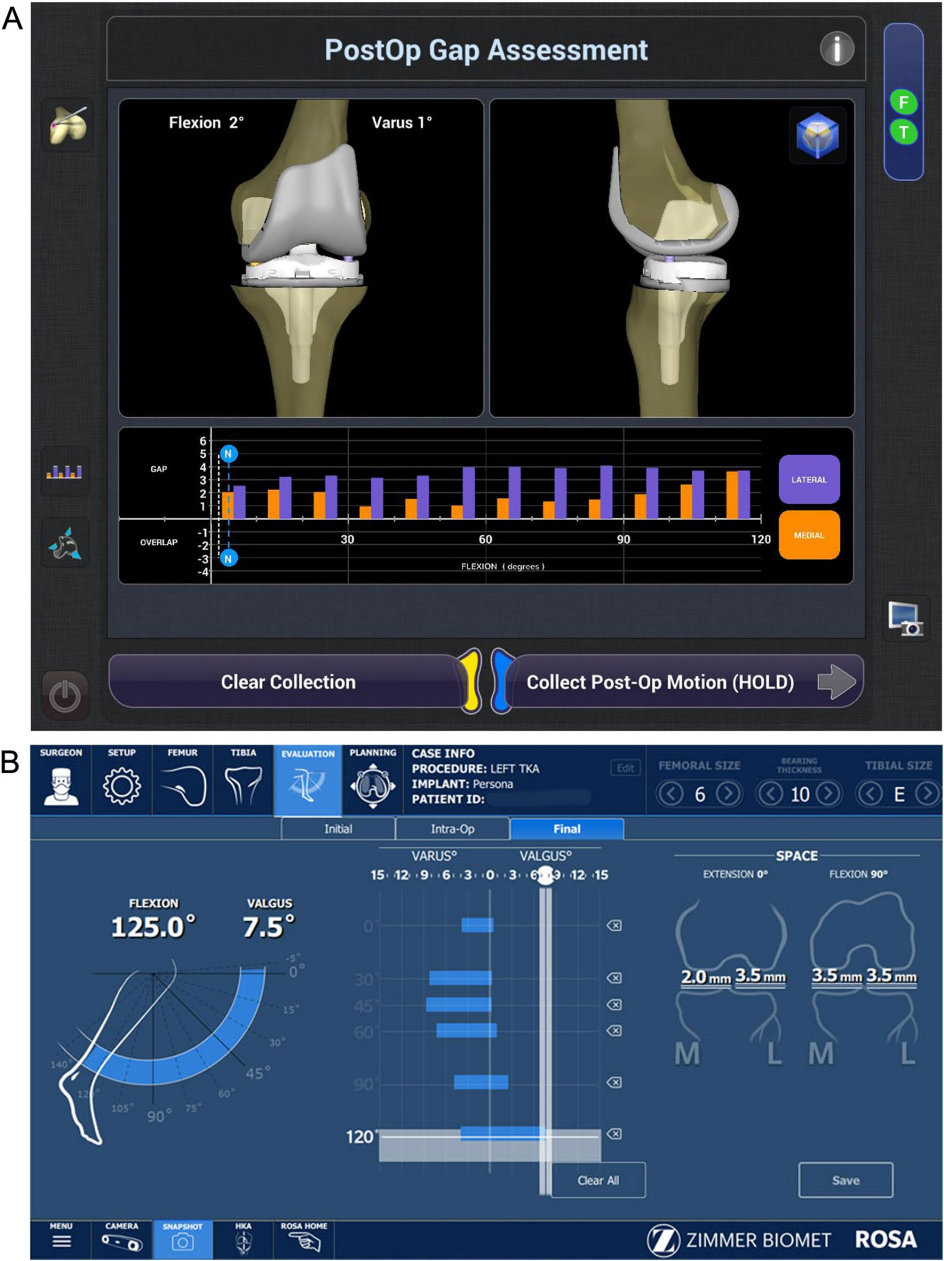
Screenshot of assessment of the soft tissue balance after implantation. (**A**) NAVIO (**B**) ROSA.

The mean intraoperative lateral laxity with the knee at 0°, 30°, 60°, 90°, and 120° was 2.8°, 2.6°, 2.9°, 3.6°, and 3.3° using NAVIO, and 2.3°, 3.5°, 3.1°, 3.2°, and 3.9° using ROSA, respectively. Lateral laxity at 30° was significantly greater in the ROSA group than in the NAVIO group (p = 0.025). The other parameters showed no significant differences. The mean intraoperative medial laxity with the knee at 0°, 30°, 60°, 90°, and 120° was 1.5°, 1.6°, 1.8°, 2.5°, and 2.9° and 1.6°, 2.4°, 1.9°, 2.6°, and 2.8°, using NAVIO and ROSA respectively. Medial laxity at 30° was significantly greater in the ROSA group than in the NAVIO group (p = 0.011). The other parameters showed no significant differences. The mean intraoperative mediolateral laxity with the knee at 0°, 30°, 60°, 90°, and 120° was 4.1°, 4.9°, 4.6°, 5.9°, and 6.1° using NAVIO (Fig. 3A) and 3.9°, 5.8°, 4.9°, 5.7°, and 6.6° using ROSA, respectively (Fig. 3B). Mediolateral laxity showed no significant differences at any of the measurements, including those at 30°. Intraoperative laxity did not affect the categories of KSS 2011 in knees operated using NAVIO and ROSA.

## Discussion

The most important findings of this study were that the two robotic systems, the image-free handheld robotic system, NAVIO, and the radiography-based robotic system, ROSA, showed no differences in the radiological alignment of the implants; however, the femoral sagittal cutting error was smaller in the NAVIO group than in the ROSA group. The possible explanations for this are as follows: (a) ROSA used a traditional bone saw, whereas NAVIO used a handheld burring device; (b) Cutting of the distal femur is likely to occur in extension due to deviation of the saw blade from the weight of the saw^21,22^. In addition, when cutting bones with sclerosis, the saw blade is likely to bend, resulting in cutting errors. (c) Cutting guide movement also leads to cutting errors, particularly in bones with osteoporosis^19^. A significant difference was observed between the average planned and validated cutting angle of the femoral flexion using ROSA robotic system, although the average difference was below 1° with standard deviation of less than 1^15^. This variance may be attributed to a potential manual error made by the surgeon when applying the validation tool to the rough bone surface.

Our results showed that in TKA, the radiography-based robotic system, ROSA resulted in a significant decrease in operative time and an increase in the KSS 2011 symptoms score compared to the image-free handheld robotic system, NAVIO. The use of a handheld burring device required more time compared to using a saw blade. The disparity in outcomes may be attributed to the implant rather than the robotic system. TKAs with the MC design have demonstrated excellent outcomes at mid-term follow-up, with no significant differences observed in case involving the preservation or sacrifice of the posterior cruciate ligament^23^.

A previous study compared the postoperative clinical scores after TKA between BCS and posterior-stabilized (PS) designs. No differences were found between the two designs in terms of pain (visual analog scale), Oxford Knee Score (OKS), or KSS 2011 at two years^24^. The first-generation BCS (Journey I, Smith & Nephew) was used in the study by Scarvell et al.^24^.

Iliotibial band (ITB) traction syndrome due to excessive lateral rollback and a risk of knee dislocation were reported using the first-generation BCS system (Journey I)^25,26^. A redesigned Journey II (Smith and Nephew) was introduced. The position of the post was moved anteriorly to further maintain a more anatomically correct femoral rollback, reducing ITB tension. The height of the posts was increased to prevent dislocation. Thus, the second-generation system (Journey II) had a lower risk of reoperation and revision than the first-generation system (Journey I)^27^. The midterm revision risk of the second-generation system (Journey II) was similar to that of the registry controls in the PS design^28^. Another study compared the second-generation BCS and PS designs, and the Knee Injury Osteoarthritis Survey for joint replacement and ROM showed no differences between the two designs^29^. When postoperative clinical scores after TKA were compared between BCS and cruciate-retaining (CR) designs, no differences were found in terms of the Western Ontario and McMaster Universities Osteoarthritis Index score and the OKS at 2 years^16^. However, the ROM was better in the BCS design than in the CR design. Indelli et al.^17^ compared the postoperative OKS, KSS 2011, and ROM after TKA between MC and PS designs. At the two-year minimum follow-up, there were no statistically significant differences between the two groups in the average OKS and KSS 2011 scores. However, ROM was better in the MC design than in the PS design. When reviewing outcomes after TKA with the use of MC, CR, or PS bearing within the same implant system, MC knees exhibited lower visual analog scale scores than PS knees at 2 weeks, 6 weeks, 3 months, and 1 year, along with a higher ROM than PS knees at 2 weeks. Additionally, MC knees demonstrated a significantly higher FJS-12 than CR knees^30^. The MC design may contribute to reduced pain in the present study. No studies have compared the early outcomes between BCS and MC designs. BCS and MC designs may affect postoperative pain.

To the best of our knowledge, the present study is the first to assess the clinical and radiographic results between NAVIO (second-generation BCS design) and ROSA (MC design) robot-assisted TKA. The BCS design is reportedly sensitive to femorotibial component rotational mismatches. The femorotibial component rotational mismatch and postoperative clinical outcomes, including flexion angle and KSS 2011 scores, were negatively correlated. The guided-motion design may have a higher joint restraint than the conventional design^31^. This rotational mismatch might be one of the reasons for the inferior KSS 2011 symptoms score in the NAVIO group.

The medial and lateral laxities at 30° were greater in knees operated using ROSA than in those using NAVIO. TKA using the BCS design can reduce mediolateral laxities in the midflexion range^32,33^. Previous studies have demonstrated that midflexion laxity is associated with patient satisfaction and expectation^32,34^. However, intraoperative laxity did not affect patient satisfaction or expectations in this study.

The present study had some limitations. First, this was a small cohort of 40 and 48 patients in the NAVIO and ROSA groups, respectively. Second, the follow-up period was only one year. Third, the femorotibial component rotational mismatch was not evaluated. Finally, both BCS (NAVIO) and MC (ROSA) designs were used in this study. Further studies should be conducted using the MC design in both robotic systems.

## Conclusions

The two robotic systems showed no differences radiographically; however, the femoral sagittal cutting error was smaller in the NAVIO group than in the ROSA group, operative time was shorter in the ROSA group, and the KSS 2011 symptoms score at one year was higher in the knees operated using ROSA than in those using NAVIO. The hypothesis that radiographic outcomes would be similar after TKA using NAVIO and ROSA was confirmed; however, another hypothesis that early clinical outcomes would be similar was disproved.

## Data Availability

The datasets generated during and/or analyzed during the current study are available from the corresponding author on reasonable request.

## References

[1] Clement ND, Al-Zibari M, Afzal I, Deehan DJ, Kader D (2020). A systematic review of imageless hand-held robotic-assisted knee arthroplasty: Learning curve, accuracy, functional outcome and survivorship. EFORT Open Rev..

[2] Batailler C, Fernandez A, Swan J, Servien E, Haddad FS, Catani F, Lustig S (2021). MAKO CT-based robotic arm-assisted system is a reliable procedure for total knee arthroplasty: A systematic review. Knee Surg. Sports Traumatol. Arthrosc..

[3] St. Mart JP, Goh EL (2021). The current state of robotics in total knee arthroplasty. EFORT Open Rev..

[4] Kort N, Stirling P, Pilot P, Müller JH (2022). Robot-assisted knee arthroplasty improves component positioning and alignment, but results are inconclusive on whether it improves clinical scores or reduces complications and revisions: A systematic overview of meta-analyses. Knee Surg. Sports Traumatol. Arthrosc..

[5] Sicat CS, Chow JC, Kaper B, Mitra R, Xie J, Schwarzkopf R (2021). Component placement accuracy in two generations of handheld robotics-assisted knee arthroplasty. Arch Orthop. Trauma Surg..

[6] Vaidya N, Jaysingani TN, Panjwani T, Patil R, Deshpande A, Kesarkar A (2022). Assessment of accuracy of an imageless hand-held robotic-assisted system in component positioning in total knee replacement: A prospective study. J. Robot Surg..

[7] Hasegawa M, Hattori Y, Naito Y, Tone S, Sudo A (2023). Comparing an imageless hand-held robotic-assisted system versus conventional technique for component positioning and early clinical outcomes in total knee arthroplasty. Int. J. Med. Robot..

[8] Matsumoto T, Nakano N, Hayashi S, Takayama K, Maeda T, Ishida K, Kuroda Y, Matsushita T, Niikura T, Muratsu H, Kuroda R (2023). Prosthetic orientation, limb alignment, and soft tissue balance with bi-cruciate stabilized total knee arthroplasty: A comparison between the handheld robot and conventional techniques. Int. Orthop..

[9] Rossi SMP, Benazzo F (2023). Individualized alignment and ligament balancing technique with the ROSA® robotic system for total knee arthroplasty. Int. Orthop..

[10] Mancino F, Rossi SMP, Sangaletti R, Caredda M, Terragnoli F, Benazzo F (2024). Increased accuracy in component positioning using an image-less robotic arm system in primary total knee arthroplasty: A retrospective study. Arch. Orthop. Trauma. Surg..

[11] Parratte S, Price AJ, Jeys LM, Jackson WF, Clarke HD (2019). Accuracy of a new robotically assisted technique for total knee arthroplasty: A cadaveric study. J. Arthroplasty..

[12] Batailler C, Hannouche D, Benazzo F, Parratte S (2021). Concepts and techniques of a new robotically assisted technique for total knee arthroplasty: The ROSA knee system. Arch. Orthop. Trauma. Surg..

[13] Batailler C, Anderson MB, Flecher X, Ollivier M, Parratte S (2023). Is sequential bilateral robotic total knee arthroplasty a safe procedure? A matched comparative pilot study. Arch. Orthop. Trauma. Surg..

[14] Mancino F, Rossi SMP, Sangaletti R, Lucenti L, Terragnoli F, Benazzo F (2023). A new robotically assisted technique can improve outcomes of total knee arthroplasty comparing to an imageless navigation system. Arch. Orthop. Trauma. Surg..

[15] Rossi SMP, Sangaletti R, Perticarini L, Terragnoli F, Benazzo F (2023). High accuracy of a new robotically assisted technique for total knee arthroplasty: An in vivo study. Knee Surg. Sports Traumatol. Arthrosc..

[16] Guta D, Santini AJ, Gornall M, Phillipson A, Davidson JS, Banks J, Pope JA, Yorke J (2023). Short-term functional comparison of three total knee arthroplasties-Journey II, Genesis II and Profix. J. Orthop. Surg..

[17] Indelli PF, Risitano S, Hall KE, Leonardi E, Migliore E (2019). Effect of polyethylene conformity on total knee arthroplasty early clinical outcomes. Knee Surg. Sports Traumatol. Arthrosc..

[18] Scuderi GR, Bourne RB, Noble PC, Benjamin JB, Lonner JH, Scott WN (2012). The new knee society knee scoring system. Clin. Orthop. Relat. Res..

[19] Hasegawa M, Tone S, Naito Y, Sudo A (2022). Two- and three-dimensional measurements following robotic-assisted total knee arthroplasty. Int. J. Med. Robot..

[20] Kanda Y (2013). Investigation of the freely available easy-to-use software 'EZR' for medical statistics. Bone Marrow Transplant..

[21] Otani T, Whiteside LA, White SE (1993). Cutting errors in preparation of femoral components in total knee arthroplasty. J. Arthroplasty..

[22] Plaskos C, Hodgson AJ, Inkpen K, McGraw RW (2002). Bone cutting errors in total knee arthroplasty. J. Arthroplasty..

[23] Rossi SMP, Sangaletti R, Jannelli E, Bova D, Montagna A, Benazzo F (2024). PCL preservation or sacrifice does not influence clinical outcomes and survivorship at mid-term follow-up of a J-curve CR total knee replacement with a medial congruent liner and a functional coronal alignment. Arch. Orthop. Trauma. Surg..

[24] Scarvell JM, Perriman DM, Smith PN, Campbell DG, Bruce WJM, Nivbrant B (2017). Total knee arthroplasty using bicruciate-stabilized or posterior-stabilized knee implants provided comparable outcomes at 2 years: A prospective, multicenter, randomized, controlled, clinical trial of patient outcomes. J. Arthroplasty..

[25] Luyckx L, Luyckx T, Bellemans J, Victor J (2010). Iliotibial band traction syndrome in guided motion TKA: A new clinical entity after TKA. Acta Orthop. Belg..

[26] Christen B, Neukamp M, Aghayev E (2014). Consecutive series of 226 journey bicruciate substituting total knee replacements: Early complication and revision rates. BMC Musculoskelet. Disord..

[27] Christen B, Kopjar B (2018). Second-generation bi-cruciate stabilized total knee system has a lower reoperation and revision rate than its predecessor. Arch. Orthop. Trauma. Surg..

[28] Harris AI, Christen B, Malcorps JJ, O'Grady CP, Kopjar B, Sensiba PR, Vandenneucker H, Huang BK, Cates HE, Hur J, Marra DA (2019). Midterm performance of a guided-motion bicruciate-stabilized total knee system: Results from the international study of over 2000 consecutive primary total knee arthroplasties. J. Arthroplasty..

[29] Shichman I, Oakley CT, Thomas J, Fernandez-Madrid I, Meftah M, Schwarzkopf R (2023). Comparison of traditional PS versus kinematically designs in primary total knee arthroplasty. Arch. Orthop. Trauma. Surg..

[30] Frye BM, Patton C, Kinney JA, Murphy TR, Klein AE, Dietz MJ (2021). A medial congruent polyethylene offers satisfactory early outcomes and patient satisfaction in total knee arthroplasty. Arthroplast. Today..

[31] Fujita M, Matsumoto T, Nakano N, Ishida K, Kuroda Y, Maeda T, Hayashi S, Kuroda R (2022). Rotational mismatch between femoral and tibial components should be avoided in JOURNEY II bi-cruciate stabilized total knee arthroplasty. Knee..

[32] Kaneko T, Kono N, Mochizuki Y, Hada M, Toyoda S, Musha Y (2017). Bi-cruciate substituting total knee arthroplasty improved medio-lateral instability in mid-flexion range. J. Orthop..

[33] Hino K, Kutsuna T, Watamori K, Ishimaru Y, Kiyomatsu H, Shiraishi Y, Miura H (2018). Bi-cruciate substituting total knee arthroplasty provides varus-valgus stability throughout the midflexion range. Knee..

[34] Hasegawa M, Naito Y, Yamaguchi T, Wakabayashi H, Sudo A (2018). Factors contributing to patient satisfaction and expectations following computer-assisted total knee arthroplasty. J. Knee Surg..

